# Lesion detection using artificial intelligence models in MR images of prostate cancer and prostatitis patients and comparison of model performance

**DOI:** 10.3389/fruro.2025.1726795

**Published:** 2026-01-19

**Authors:** Muhammed Kaya, Osman Durdag, Merve Solak, Ahmet Coskuncay, Gulen Burakgazi

**Affiliations:** 1 Faculty of Medicine, Recep Tayyip Erdoğan University, Rize, Türkiye; 2 Department of Software Engineering, Ataturk University, Erzurum, Türkiye; 3 Department of Computer Engineering, Ataturk University, Erzurum, Türkiye

**Keywords:** artificial intelligence, faster R-CNN, magnetic resonance imaging, prostatic neoplasms, prostatitis

## Abstract

**Aim:**

The diagnosis of prostate cancer and prostatitis becomes challenging when using biparametric Magnetic Resonance (MR) images. This research investigates deep learning models to assess their capability for improving diagnostic accuracy and assisting radiologists.

**Methods:**

This retrospective study analyzed 153 patients who received histopathological diagnoses of prostate cancer or prostatitis between January 2017 and December 2023. Patients were categorized according to PI-RADS scores, and both T2A and ADC-DWI (Apparent Diffusion Coefficient–Diffusion-Weighted Imaging) sequences were examined. Expert radiologists labeled the images prior to lesion detection with the Faster R-CNN (Faster Region-based Convolutional Neural Network) model. Nine different classification models were trained using normal and augmented datasets to evaluate their performance. Model reliability was further assessed through cross-validation and statistical significance testing.

**Results:**

The Faster R-CNN model achieved 96% accuracy (95% CI: 93.2–98.8%) for P5 and 99% accuracy (95% CI: 96.7–100%) for prostatitis in T2A sequences, and 90% accuracy (95% CI: 85.4–94.6%) for P5 and 97% accuracy (95% CI: 93.8–100%) for prostatitis in ADC-DWI sequences. However, the model failed to effectively detect P4 lesions (0% sensitivity in T2A and 30% in ADC-DWI). The model demonstrated comparable performance to expert radiologists, with no significant difference in overall P5 detection (p > 0.05), and Cohen’s kappa indicated substantial agreement (κ = 0.86). The classification models achieved up to 97% accuracy with InceptionV3 in T2A sequences and up to 99% accuracy with DenseNet201 in ADC-DWI sequences. To further evaluate discriminative performance, AUROC values were calculated for all classification models. In T2A sequences, AUROC scores were DenseNet201 (0.98), EfficientNetV2L (0.99), InceptionV3 (0.99), MobileNetV2 (0.92), NASNetLarge (0.83), ResNet50 (0.76), VGG16 (0.98), VGG19 (0.97), and Xception (0.96). In ADC-DWI sequences, AUROC values were DenseNet201 (0.99), EfficientNetV2L (0.96), InceptionV3 (0.99), MobileNetV2 (0.82), NASNetLarge (0.90), ResNet50 (0.64), VGG16 (0.96), VGG19 (0.86), and Xception (0.97), reinforcing the superior discriminative ability of DenseNet201 and InceptionV3 across modalities.

**Conclusion:**

The deep learning models demonstrated promising diagnostic capabilities, comparable to radiologists, in distinguishing prostatitis and P5 prostate cancer lesions. Overall, the findings suggest that AI-based diagnostic tools hold potential as clinical decision support systems.

## 1 Introduction

Prostate cancer stands as the second most fatal type of cancer that affects men (1, 2). The clinical challenge of accurate prostate cancer diagnosis has driven the evolution of diagnostic approaches from traditional methods to advanced imaging and artificial intelligence-assisted techniques.

The diagnosis of prostate cancer relies on three main methods which include prostate-specific antigen (PSA) testing and digital rectal examination (DRE) and systematic prostate biopsy under transrectal ultrasound (TRUS) guidance (3–6). The glycoprotein PSA functions as a biomarker but shows poor cancer specificity because it originates from prostate epithelial cells. Serum PSA levels increase in patients with BPH and prostatitis and after prostate biopsy procedures because this test lacks sufficient cancer specificity (6–8). The diagnostic value of PSA measurements is restricted since histopathological confirmation of PCa through TRUS-guided biopsy occurs in less than half of patients (8–10). The anatomical coverage limitations and random systematic biopsy approach of TRUS produce high false-negative rates (11, 12).

The development of MRI technology has revolutionized the process of diagnosing prostate cancer. The diagnostic capabilities of MRI significantly improved through the addition of functional imaging sequences including diffusion-weighted imaging (DWI) and dynamic contrast-enhanced MRI (DCE-MRI) in the late 1990s, leading to enhanced PCa detection (13, 14). The combination of these techniques led to the development of multiparametric MRI (mpMRI) in the 2000s, enabling detailed anatomical and functional examination of prostate tissue using T2-weighted imaging, DWI, DCE-MRI, and, when necessary, magnetic resonance spectroscopy (MRS) sequences (15).

Since the 2010s, mpMRI has become the standard imaging method for the diagnosis, local staging, and treatment planning of PCa (16). Structured reporting systems, particularly the Prostate Imaging Reporting and Data System (PI-RADS), were developed to improve both diagnostic precision and reporting uniformity in mpMRI assessments (17–19). Currently, mpMRI serves as the primary non-invasive imaging technique for both selecting biopsy locations and guiding biopsy targets (19).

Medical imaging has experienced a transformative shift through artificial intelligence (AI) which enhances both diagnostic precision and operational speed. AI applications have shown significant advancement in prostate cancer diagnosis through automated image analysis and pattern recognition and clinical decision support systems (20). Research shows that deep learning algorithms specifically convolutional neural networks (CNNs) achieve superior results in complex imaging data analysis by matching or surpassing the diagnostic abilities of trained radiologists (20).

The current developments in AI-assisted prostate MRI analysis include automated lesion detection, segmentation, and characterization. The systems use different deep learning architectures such as U-Net, Mask R-CNN, and Faster R-CNN for different analytical tasks (21). The implementation of these deep learning-based algorithms has demonstrated high accuracy in performing lesion segmentation, PI-RADS scoring, and malignancy prediction (21–23). These systems demonstrate potential to decrease inter-reader variability while enhancing diagnostic accuracy (21). The technology demonstrates potential for differentiating prostate cancer from benign conditions which represents a major diagnostic obstacle (24).

The literature contains multiple essential gaps despite recent progress. The majority of research studies detect cancer but fail to distinguish between cancerous and benign conditions. The majority of AI models were developed and validated using limited datasets from individual institutions which creates doubts about their ability to generalize. The current evidence base lacks sufficient data to compare AI diagnostic accuracy with that of experienced radiologists in actual clinical settings (21, 24).

In this study, deep learning-based artificial intelligence models were used to automatically detect prostate lesions and perform zonal localization (transitional zone [TZ] and peripheral zone [PZ]) with high accuracy using biparametric MR (bpMR) images from patients with histopathologically confirmed PCa or prostatitis. The research focuses on developing precise methods to distinguish prostate cancer from prostatitis because this distinction determines important clinical treatment choices. The diagnostic performance of the developed models was compared with visual evaluations by experienced radiologists to scientifically demonstrate the contribution of AI-supported analysis compared to classical interpretation methods based on human observation. The research uses advanced deep learning architectures together with thorough validation methods to establish strong evidence about AI utility in prostate imaging. In this context, the feasibility of AI as a decision support tool in prostate imaging was evaluated, and its potential for clinical integration was explored.

## 2 Methods

### 2.1 Study design and sample size calculation

The research followed STARD (Standards for Reporting of Diagnostic Accuracy Studies) guidelines in its retrospective cohort study design. The G*Power 3.1 software performed power analysis to establish the smallest necessary sample size. The literature shows AI-based prostate cancer detection achieves sensitivity rates between 85-90% so we used 90% sensitivity and 85% specificity with a 60% disease prevalence in our biopsy-confirmed population at an alpha error of 0.05 and power of 0.80 to calculate a minimum sample size of 138 patients. The research included 153 patients which exceeded the calculated minimum sample size for sufficient statistical power.

### 2.2 Study population and patient selection criteria

The research examined patients with confirmed prostate cancer or prostatitis diagnoses at our hospital from January 2017 to December 2023. The study included participants who met the following criteria: (1) aged 45–85 years; (2) histopathological confirmation of prostate cancer or prostatitis through systematic 12-core TRUS-guided biopsy or MRI-targeted biopsy performed within 6 weeks of MRI; (3) complete biparametric MRI sequences (T2-weighted and DWI/ADC); (4) no prior prostate surgery, radiation therapy, or hormonal treatment; (5) PSA levels between 2.5–50 ng/mL at the time of MRI. The exclusion criteria consisted of: (1) MRI sequences that were either incomplete or motion-degraded with artifact score >2 on a 5-point scale; (2) hip prosthesis or other metallic implants that caused significant susceptibility artifacts; (3) the time interval between MRI and biopsy was more than 6 weeks; (4) other malignancies were present; (5) insufficient histopathological sampling (<10 cores for systematic biopsy). The study included 153 patient images from a total of 180 patients because 23 patients failed exclusion criteria and 4 patients had lost or corrupted data before model training.

#### 2.2.1 Important clarification

PI-RADS scores were used for risk stratification and initial categorization, not as definitive diagnoses. All cases were confirmed through histopathological examination. For analysis purposes, histopathologically confirmed prostate cancer lesions smaller than 1.5 cm were labeled as P4, whereas those larger than 1.5 cm were labeled as cancer. Lesions histopathologically confirmed as prostatitis were labeled as prostatitis. In the T2 sequence, lesions diagnosed as prostatitis and categorized under the P3 label comprise 277 training images and 71 test images. Among the 1,906 training images labeled as prostate cancer, only 65 were assigned to the P4 category, while the remaining 1,841 were classified as P5; similarly, of the 488 test images, 13 were labeled as P4 and 475 as P5. In the ADC–DWI sequence, prostatitis cases corresponding to the P3 label include 267 training and 69 test images. Within this modality, of the 1,965 training images associated with prostate cancer, only 95 were categorized as P4 and 1,870 as P5; likewise, among the 489 test images, 19 were assigned to the P4 class and 470 to the P5 class However, due to the insufficient number of P4-labeled samples—resulting in limited statistical power and suboptimal model performance—and considering that the P4 label inherently denotes cancer, the P4 and P5 categories were consolidated and treated as a single cancer class during the classification stages of the study. This approach ensures that our model training was based on ground truth pathology results rather than imaging scores alone.

### 2.3 Imaging protocol and data acquisition

All patients underwent biparametric MR imaging using a standardized protocol on a 3.0 Tesla MR device (GE Discovery MR750w, GE Healthcare, Waukesha, Wisconsin, USA) with a 32-channel phased-array body coil. Standard imaging protocols included T2-weighted turbo spin-echo scans with TR = 4068 ms, TE = 130 ms, field of view=180×180 mm, matrix=320×320, and slice thickness of 3.5 mm with no gap, and diffusion-weighted echo-planar imaging scans with TR = 4600 ms, TE = 90 ms, field of view=240×240 mm, matrix=128×128, and slice thickness of 4 mm at b50, b800, and b1400 values. ADC maps were automatically generated using monoexponential fitting.

### 2.4 Image annotation and inter-rater reliability

**Figure 1:**
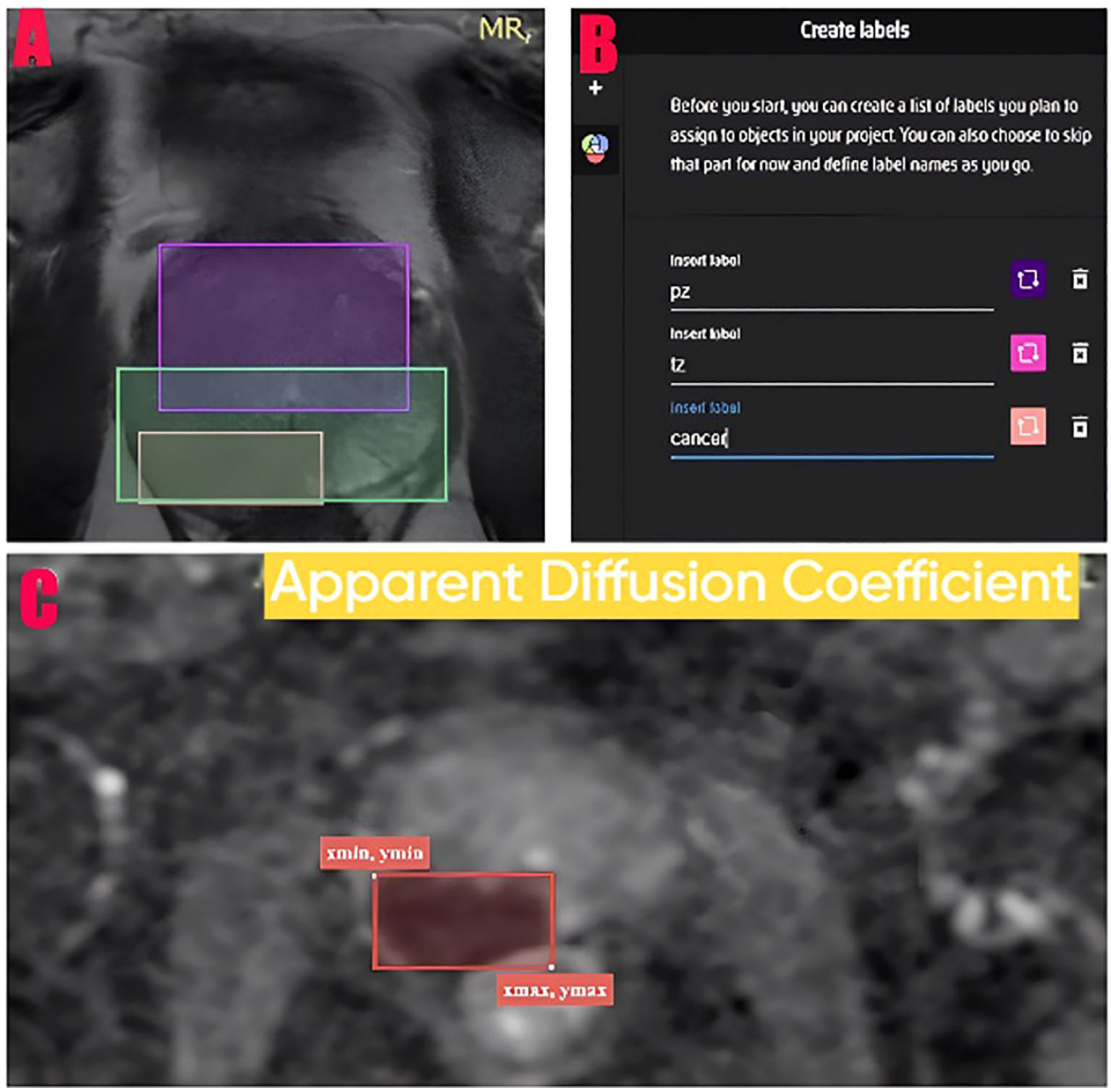
Data labeling methodology and coordinate system. **(A)** The axial T2 MR image displays multiple labels for peripheral zone (pz - purple), transitional zone (tz - pink) and cancer (cancer - orange). **(B)** The MakeSense platform interface and label creation panel. **(C)** The ADC image shows a coordinate system which displays the xmin, ymin (top left corner) and xmax, ymax (bottom right corner) coordinates of the label. The image coordinate system begins at the top left corner (0,0) and extends to the right and downwards.

The entire imaging dataset underwent evaluation by two specialized radiologists with 3 and 5 years of prostate MRI experience, working independently and blinded to histopathological results. Final verification was performed by a senior radiologist who has practiced for 15 years to resolve discrepancies. The reliability between raters was evaluated through Cohen’s kappa coefficient for p4, p5(cancer) and prostatitis (κ=0.82, indicating substantial agreement) and intraclass correlation coefficient (ICC) for lesion measurements (ICC = 0.89, 95% CI: 0.84-0.93, indicating excellent reliability). The data collection tool reliability and validity was confirmed through MakeSense platform labeling and XML format coordinate recording with experienced radiologist verification (**Figure 1**). All annotations were performed according to PI-RADS v2.1 guidelines (25).

### 2.5 Image processing and data augmentation

**Figure 2:**
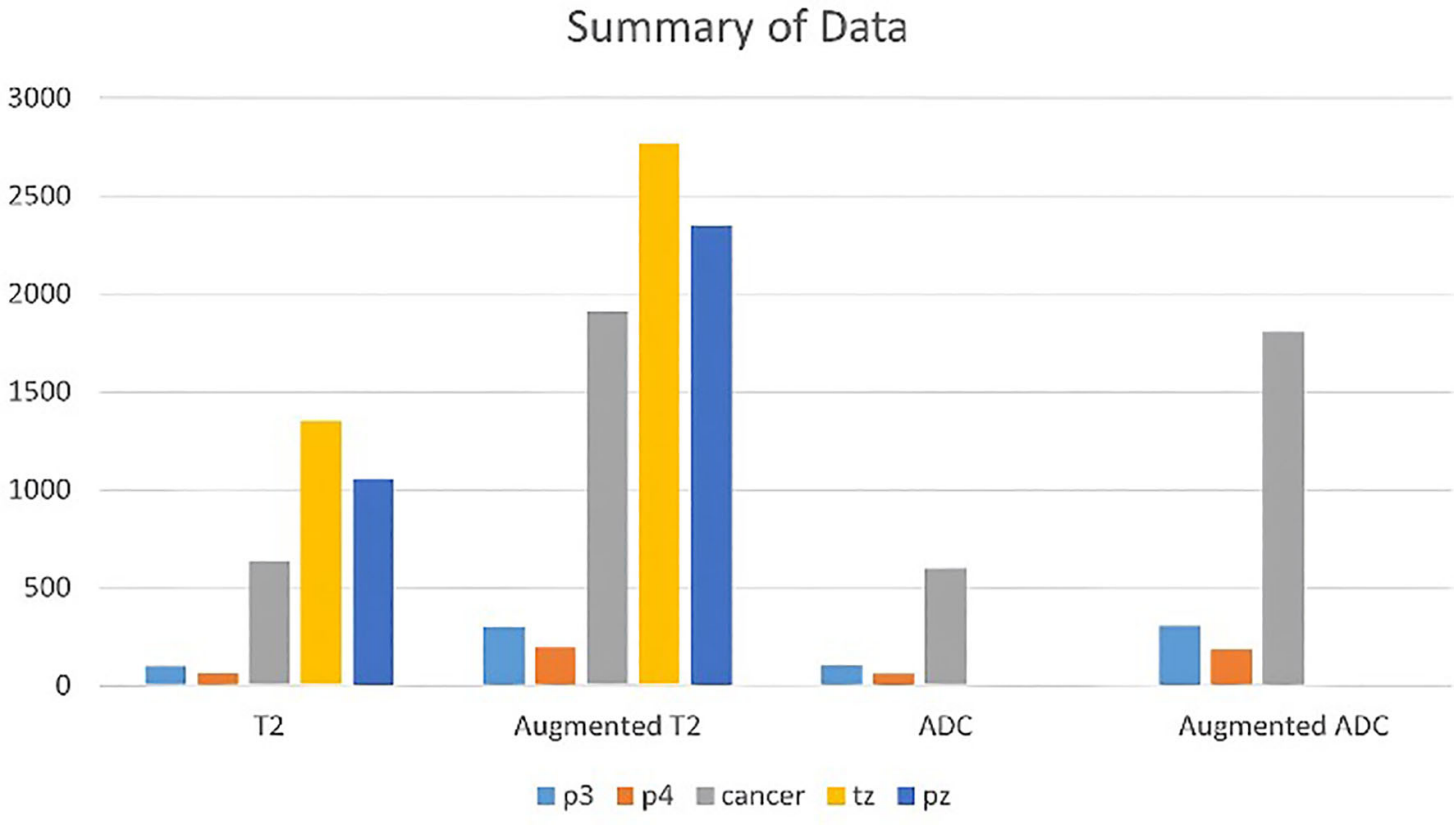
A chart summarizing the dataset composed of T2 and ADC images, along with the corresponding augmented dataset.

Standardization of T2 images involved transforming various resolution levels into 512×512 pixels using bilinear interpolation, followed by ADC image standardization to 256×256 pixels. Images were normalized to zero mean and unit variance. The OpenCV library’s convertScaleAbs function was applied to data augmentation protocols for image processing through two sets of parameters (alpha=2, beta=0 and alpha=1.5, beta=25) to enhance contrast and brightness variations. The dataset received additional augmentation through random rotation (± 15 degrees) and horizontal flipping (probability=0.5). The images generated through the data augmentation process were reviewed by expert radiologists, who confirmed that no distortions or alterations occurred in the morphology of the prostate tissue. The summary of the dataset obtained through data augmentation is presented in **Figure 2**.

### 2.6 Deep learning model architecture

#### 2.6.1 Faster R-CNN architecture details

The Faster R-CNN model architecture consisted of a ResNet-50 backbone pre-trained on ImageNet, a Region Proposal Network (RPN) with 3×3 convolutional layers, and a Fast R-CNN detector head. The RPN used three anchor scales (128², 256², 512²) and three aspect ratios (1:2, 1:1, 2:1) at each spatial position. Non-maximum suppression with IoU threshold of 0.7 was applied to reduce redundant detections. The model received integration through Facebook AI Research’s detectron2 library version 0.6 during the model training protocol. The training hyperparameters consisted of a learning rate set to 0.001 with step decay at epochs 60 and 80, momentum set to 0.9, weight decay set to 0.0001, batch size set to 4, and the number of epochs was set to 90 with early stopping patience of 10 epochs based on validation loss. Training was performed on the Google Colab platform using NVIDIA Tesla P100 GPU with 16GB memory.

#### 2.6.2 Classification model architecture

The classification models employed transfer learning with ImageNet pre-trained weights for nine architectures: InceptionV3, Xception, DenseNet201, EfficientNetV2L, VGG16, VGG19, NASNetLarge, MobileNetV2, and ResNet50. All models were modified by removing the original classification head and adding new final layers with a Global Average Pooling + Dropout (rate=0.5) + Dense (256 units, ReLU activation) + Dropout (rate=0.3) + Dense (2 units) + Softmax structure. Fine-tuning was performed on the last 30% of layers while keeping earlier layers frozen for the first 20 epochs, then unfreezing all layers for continued training.

### 2.7 Model training and validation strategy

A stratified splitting approach ensured balanced representation of both classes across datasets. The training-testing ratio used for Faster R-CNN was 80% for training and 20% for testing, while the classification models received 70% for training and 15% for both validation and testing. The model stability and generalizability was assessed by performing 5-fold stratified cross-validation. Each fold had the same class distribution as the full dataset. The performance metrics were averaged across all folds with standard deviations reported.

### 2.8 Statistical analysis

Python programming language version 3.9 with scikit-learn 1.0.2, TensorFlow 2.8.0, and statsmodels 0.13.2 libraries served for statistical analysis to calculate model performance through accuracy, precision, recall (sensitivity), specificity, and F1-score values. The evaluation metrics included area under the receiver operating characteristic curve (AUC-ROC), area under the precision-recall curve (AUC-PR), and Matthews correlation coefficient (MCC).

The chi-square test evaluated categorical data while the Student’s t-test examined continuous variables for normally distributed data, with Mann-Whitney U test used for non-parametric data. A P-value of <0.05 established statistical significance in all comparative analyses, with Bonferroni correction applied for multiple comparisons. Cohen’s kappa coefficient assessed radiologist-artificial intelligence model agreement. The analysis excluded images with missing labels, and the researchers checked data consistency through automated scripts and manual verification. The study evaluated T2 and ADC-DWI sequence performance separately while comparing outcomes between normal and enhanced data sets. The confusion matrix enabled model classification success visualization, and ROC analysis found the optimal threshold values using Youden’s index. Bootstrap resampling (n=1000) was used to calculate 95% confidence intervals for all performance metrics.

## 3 Results

### 3.1 Patient characteristics and dataset description

In our study, a total of 153 patients were analyzed, and 27 of the 180 patients initially evaluated were excluded from the study due to various exclusion criteria. The patients’ biparametric MR images were obtained using a 3.0 Tesla MR device, and T2-weighted sequences and diffusion-weighted imaging techniques were applied. A total of 2,742 images from T2 sequences and 2,790 images from ADC-DWI sequences were used for the Faster R-CNN model. Two different datasets were prepared for training the classification models, one with normal images and one with augmented data, with cancer and prostatitis-diagnosed images grouped separately (**Table 1**).

**Table 1:** Patient demographics and dataset characteristics.

Parameter	Value	95% CI
Number of Patients		
Initial Assessment	180 patients	–
Excluded Patients	27 patients (23 clinical + 4 technical)	–
Final Analysis	153 patients	–
Mean Age (years)	62.4 ± 8.7	[60.0-64.8]
Mean PSA (ng/mL)	11.3 ± 6.2	[9.8-12.8]
Study Period	January 2017 – December 2023	–
Imaging Protocol		
MRI Scanner	3.0 Tesla (GE Discovery MR750w)	–
Sequences Used	T2A + DWI-ADC (Biparametric)	–
MRI Parameters		
T2A: TR/TE	4068/130 ms	–
T2A Slice Thickness	3.5 mm	–
DWI: b-values	b50, b800, b1400	–
DWI Slice Thickness	4 mm	–
Histopathological Distribution		
Prostate Cancer (n)	102 (66.7%)	[58.9-74.5%]
Prostatitis (n)	51 (33.3%)	[25.5-41.1%]
Total Faster R-CNN Images		
T2 (Training + Test)	2,742 images (2,194 + 548)	–
ADC-DWI (Training + Test)	2,790 images (2,232 + 558)	–
Data Distribution in Classification Models		
T2 Normal (Train/Val/Test)	800 (560/120/120) images	–
- Cancer/Prostatitis	701/99 images	–
T2 Augmented (Train/Val/Test)	2,400 (1,680/360/360) images	–
- Cancer/Prostatitis	2,103/297 images	–
ADC Normal (Train/Val/Test)	766 (536/115/115) images	–
- Cancer/Prostatitis	704/62 images	–
ADC Augmented (Train/Val/Test)	2,298 (1,608/345/345) images	–
- Cancer/Prostatitis	1,992/306 images	–

CI, confidence interval; PSA, prostate-specific antigen; MRI, magnetic resonance imaging; DWI, diffusion-weighted imaging; ADC, apparent diffusion coefficient; TR/TE, repetition time/echo time. Values are presented as mean ± standard deviation or n (%) where appropriate.

### 3.2 Faster R-CNN model performance

The performance evaluation of the Faster R-CNN model resulted in 96% accuracy (95% CI: 93.2-98.8%), 96% precision, and 98% F1-score for P5 lesions in T2-weighted images. In the prostatitis category, which includes prostatitis lesions, the model achieved 99% accuracy (95% CI: 96.7-100%) and 91% F1 score. The model failed to detect P4 lesions in T2 sequences because it misclassified all P4 cases as prostatitis or P5 (sensitivity = 0%). In ADC-DWI sequences, the model achieved 90% accuracy (95% CI: 85.4-94.6%) and 94% F1 score for P5 lesions, and 97% accuracy (95% CI: 93.8-100%) and 88% F1 score for prostatitis lesions. In the P4 category, low performance was again observed, with only 30% sensitivity and 43% F1 score values calculated (p<0.001 compared to prostatitis and P5 categories) (**Table 2**).

**Table 2:** Faster R-CNN model performance metrics with statistical significance.

Sequence	PI-RADS Score	Accuracy	Precision	Recall	F1-Score	95% CI (F1)	p-value
T2A Sequences							
	P5 (Cancer)	0.96	0.96	1.00	0.98	[0.95-1.00]	Reference
	P4	0.97	0.00*	0.00*	0.00*	N/A	<0.001
	Prostatitis	0.99	1.00	0.83	0.91	[0.84-0.98]	0.082
ADC-DWI Sequences							
	P5 (Cancer)	0.90	0.90	0.99	0.94	[0.90-0.98]	Reference
	P4	0.93	0.75	0.30	0.43	[0.28-0.58]	<0.001
	Prostatitis	0.97	1.00	0.79	0.88	[0.81-0.95]	0.124
Test Set Size							
	T2A	72 images					
	ADC-DWI	114 images					

*PI-RADS, Prostate Imaging Reporting and Data System; CI, confidence interval; N/A, not applicable. Model failed to detect any P4 lesions in T2A sequences. P-values calculated using chi-square test comparing to P5 category performance.

### 3.3 Comparison with radiologist performance

The artificial intelligence model achieved high agreement rates when compared to an experienced radiologist (Cohen’s κ = 0.86, 95% CI: 0.79-0.93, p<0.001). The artificial intelligence model correctly identified zonal anatomical localization in T2-weighted images at a 98.1% rate except for one image that misclassified a peripheral zone lesion as a transitional zone. The PI-RADS scoring results showed high agreement between the radiologist and artificial intelligence model with rates between 92% and 96%. The artificial intelligence model achieved 100% accuracy in cancer case classification which exceeded the radiologist’s 97.9% accuracy rate, though this difference was not statistically significant (p=0.314, McNemar’s test). The prostatitis lesions in ADC-DWI sequences received 100% agreement, but no significant statistical difference emerged between the two evaluators in the overall assessment (**Table 3**).

**Table 3:** Comparison of radiologist versus artificial intelligence with statistical analysis.

Evaluation parameter	Radiologist	AI Model	Agreement	κ (95% CI)	p-value
T2A Zonal Localization (n=52)				0.96 [0.89-1.00]	<0.001
Transitional Zone (TZ)	14 lesions	15 lesions	98.1%		
Peripheral Zone (PZ)	38 lesions	37 lesions	98.1%		
Misclassification	–	1 PZ→TZ	–		0.317
T2A PI-RADS Scoring (n=52)				0.89 [0.80-0.98]	<0.001
Prostatitis	5 lesions	4 lesions	96.2%		
P4	4 lesions	2 lesions	92.3%		
P5 (Cancer)	43 lesions	46 lesions	94.2%		
True Cancer Cases (n=48)					
Correct Classification	47/48 (97.9%)	48/48 (100%)	97.9%	0.98 [0.94-1.00]	0.314
Misclassification	1 cancer→prostatitis	0	–		
ADC-DWI PI-RADS Scoring (n=87)				0.91 [0.84-0.98]	<0.001
Prostatitis	15 lesions	15 lesions	100%		
P4	14 lesions	10 lesions	88.5%		
P5 (Cancer)	58 lesions	62 lesions	93.1%		
Overall Performance				0.86 [0.79-0.93]	<0.001

AI, artificial intelligence; TZ, transitional zone; PZ, peripheral zone; PI-RADS, Prostate Imaging Reporting and Data System; κ, Cohen’s kappa coefficient; CI, confidence interval. P-values calculated using McNemar’s test for paired comparisons.

**Figure 3:**
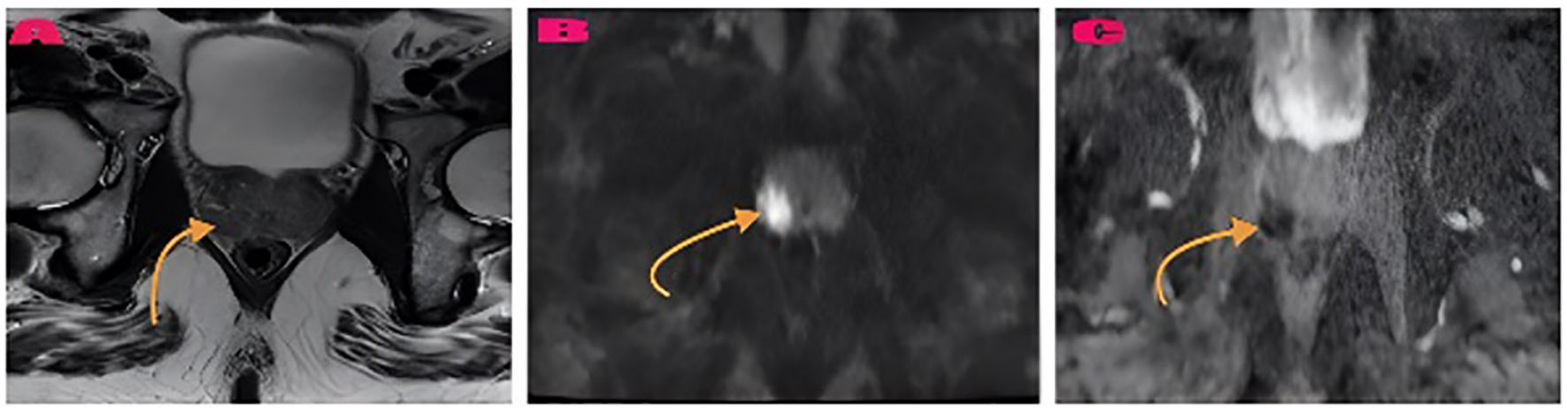
PIRADS 5 peripheral zone lesion and AI-compatible classification example. **(A)** A right peripheral zone contains an abnormal hypointense lesion which appears in an axial T2-weighted image (arrow). **(B)** Hyperintense signal with restriction on DWI b1400. **(C)** The ADC map shows a hypointense area with a low ADC value (0.56 × 10^−3^ mm^2^/s) (arrows). The radiologist and AI model agreed that this lesion should be classified as PIRADS 5. Histopathology: prostate adenocarcinoma.

**Figure 4:**
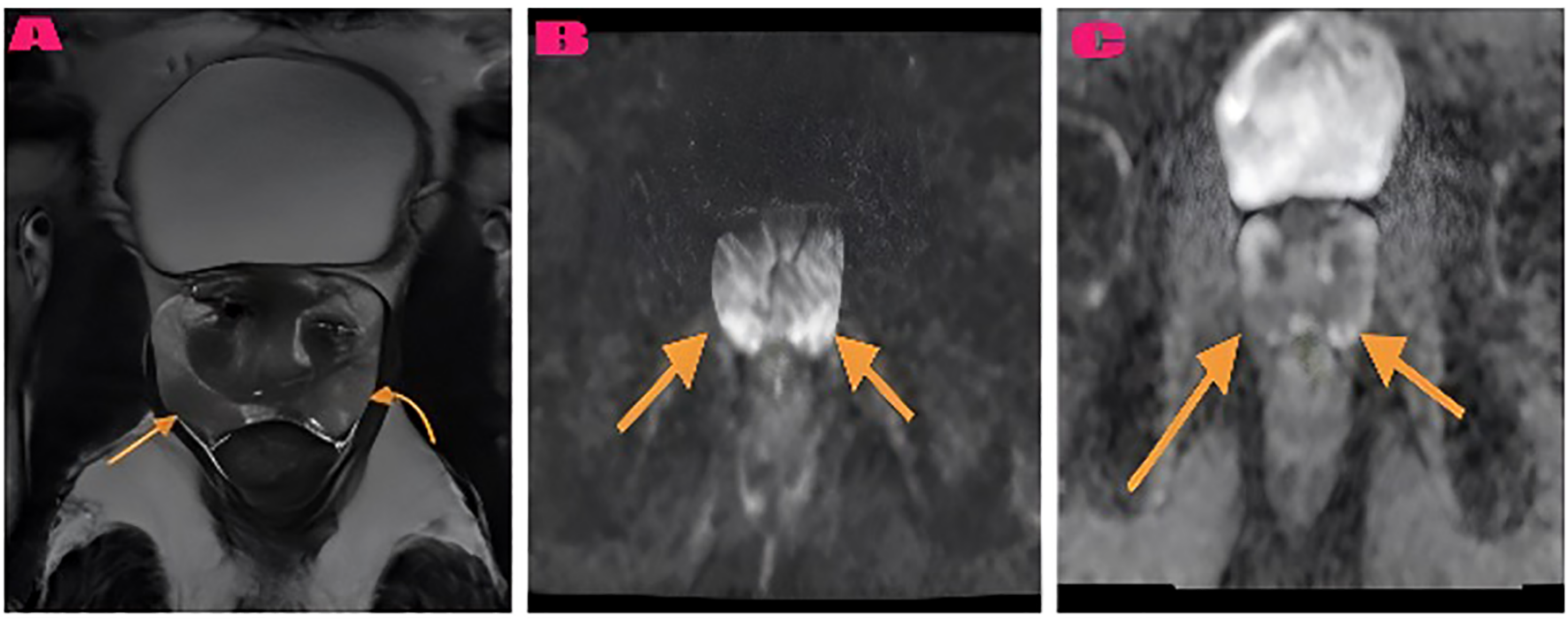
The Prostatitis bilateral peripheral zone lesion and AI-compatible prostatitis diagnosis. **(A)** The bilateral peripheral zone displays multiple hypointense lesions in axial T2-weighted imaging (arrows). **(B)** The DWI b1400 images show minimal diffusion restriction. **(C)** The ADC map shows high ADC values (0.96 × 10^−3^ mm^2^/s) in the areas marked by arrows. The radiologist and artificial intelligence model both agreed on prostatitis classification. Histopathology: chronic prostatitis.

**Figure 5:**
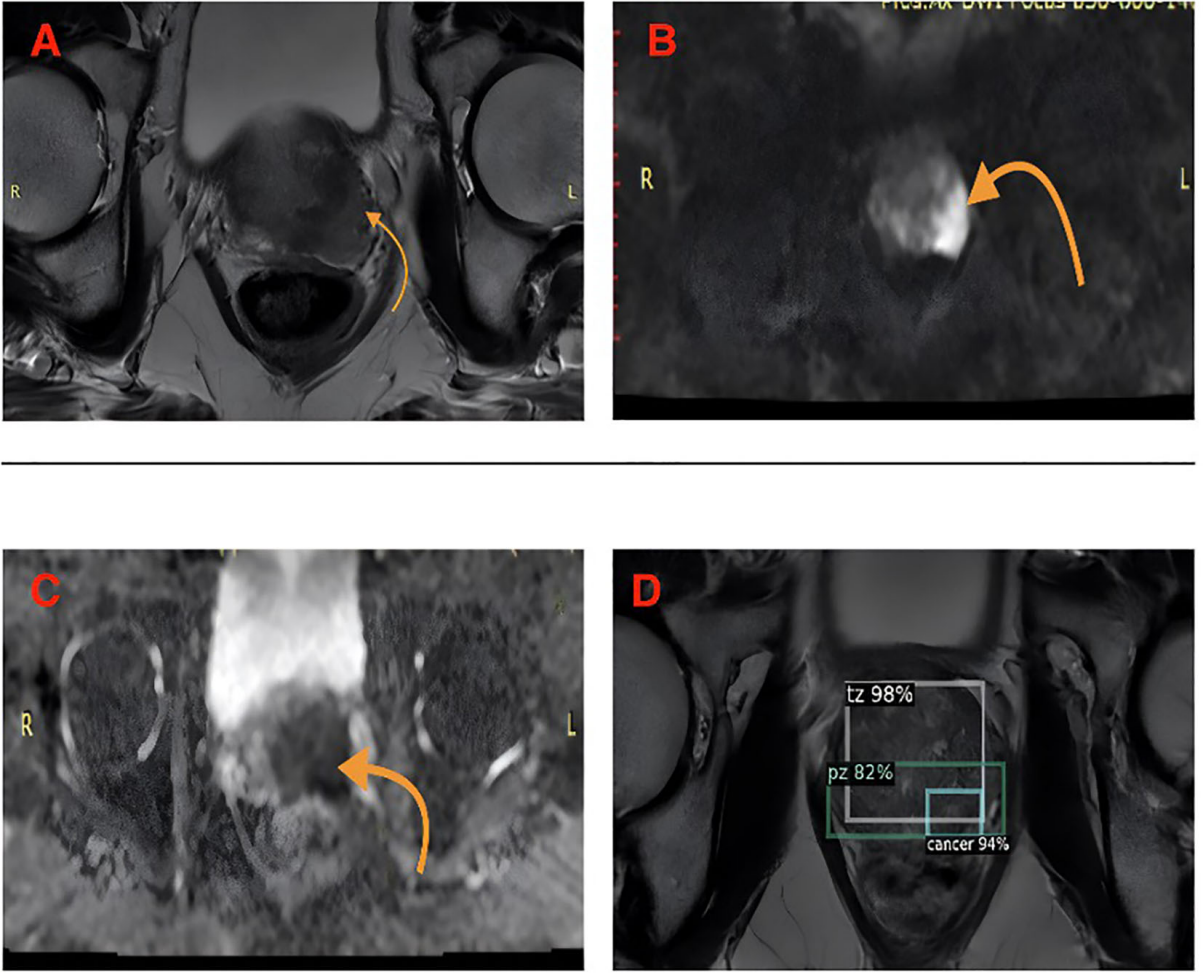
AI-based probability estimates in a patient with a PIRADS 5 lesion. **(A)** Axial T2-weighted image shows an irregularly marginated hypointense lesion larger than 15 mm located in the left peripheral zone at the prostatic apex (arrow). **(B)** The lesion appears hyperintense on high b-value diffusion-weighted imaging (b1400) (arrow). **(C)** The ADC map reveals a hypointense area with a low mean ADC value of 0.68 × 10^−3^ mm^2^/s, consistent with restricted diffusion. **(D)** Both the radiologist and the AI model assigned a PIRADS 5 score based on T2WI and DWI features. Histopathological diagnosis confirmed prostate adenocarcinoma.

PIRADS 5 peripheral zone lesion and AI-compatible classification example shown in **Figure 3**. The radiologist and AI model agreed that this lesion should be classified as PIRADS 5. Histopathology confirmed the diagnosis as prostate adenocarcinoma. As another example, a bilateral peripheral zone prostatitis lesion and the corresponding AI-consistent prostatitis diagnosis are presented in **Figure 4**. Both the radiologist and the AI model concurred in the classification of the lesion as prostatitis. Histopathology confirmed the diagnosis as chronic prostatitis. As another example, AI-based probability estimations for a patient with a PIRADS 5 lesion are presented in **Figure 5**. Both the radiologist and the AI model assigned a PIRADS 5 score based on the T2WI and DWI characteristics. The histopathological diagnosis confirmed the presence of prostate adenocarcinoma.

### 3.4 Confusion matrix analysis

**Figure 6:**
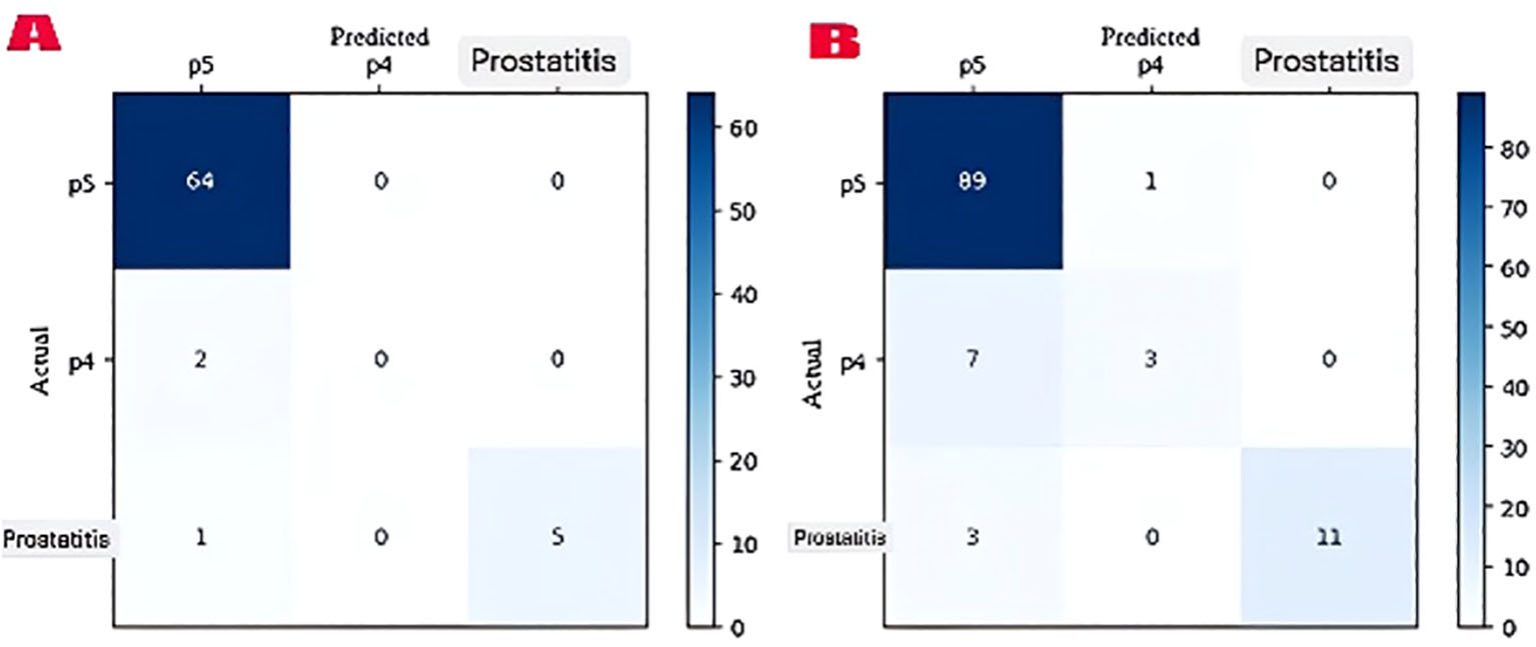
Faster R-CNN model confusion matrices. **(A)** T2-weighted images: The model correctly identified 64/67 P5 lesions together with 5/6 prostatitis lesions yet it failed to detect any P4 lesions (0/2). **(B)** ADC-DWI images: The model reached 89/90 P5 accuracy and 3/10 P4 accuracy and 11/14 prostatitis accuracy. The actual case numbers are represented by numbers while correct predictions appear on the diagonal.

Confusion matrix analyses have revealed the classification performance of the model in detail. In T2-weighted images, the model correctly classified 64 of the 72 test images, successfully identifying 5 prostatitis lesions. However, no correct predictions were made in the P4 class. In ADC-DWI images, out of 114 test images, 89 P5 lesions, 3 P4 lesions, and 11 prostatitis lesions were correctly classified (overall accuracy: 90.4%, 95% CI: 84.7-96.1%) (**Figure 6**).

### 3.5 Classification model performance and overfitting analysis

The evaluation of nine classification models showed that data augmentation led to better performance results for all models. We conducted extra validation through cross-validation and learning curve analysis to address potential overfitting issues. The InceptionV3, Xception, DenseNet201, and EfficientNetV2L models achieved 100% values in all metrics after data augmentation in T2 sequences. Although the cross-validation metrics for the other models did not reach perfect performance, they still demonstrated improved results following the data augmentation process. The lowest performance in the normal data set was recorded by the ResNet50 model with 82.5% accuracy. In the ADC-DWI sequences, the DenseNet201 model showed the highest performance with 99.1% accuracy even in the normal data set and achieved excellent results after data augmentation. The learning curves showed that the model converged well without any significant difference between training and validation performance after epoch 60 (**Table 4**). In addition to these metrics, AUROC values were calculated to further assess the discriminative capability of the classification models. For the T2 sequence, AUROC scores were as follows: DenseNet201 (0.98), EfficientNetV2L (0.99), InceptionV3 (0.99), MobileNetV2 (0.92), NASNetLarge (0.83), ResNet50 (0.76), VGG16 (0.98), VGG19 (0.97), and Xception (0.96). For the ADC-DWI sequence, AUROC values were DenseNet201 (0.99), EfficientNetV2L (0.96), InceptionV3 (0.99), MobileNetV2 (0.82), NASNetLarge (0.90), ResNet50 (0.64), VGG16 (0.96), VGG19 (0.86), and Xception (0.97), further confirming the superior discrimination performance of DenseNet201 and InceptionV3 across both modalities.

**Table 4:** Classification model performance with cross-validation results.

Sequence	Model	Normal dataset	Prec	Rec	F1	Augmented dataset	Prec	Rec	F1	CV accuracy
		Acc				Acc				Mean ± SD
T2 Sequences										
	InceptionV3	0.966	0.972	0.959	0.965	1.000	1.000	1.000	1.000	0.968 ± 0.021
	Xception	0.950	0.956	0.943	0.949	1.000	1.000	1.000	1.000	0.964 ± 0.024
	DenseNet201	0.958	0.964	0.951	0.957	1.000	1.000	1.000	1.000	0.971 ± 0.019
	EfficientNetV2L	0.941	0.948	0.934	0.941	1.000	1.000	1.000	1.000	0.962 ± 0.026
	VGG16	0.900	0.908	0.891	0.899	0.997	0.997	0.997	0.997	0.948 ± 0.032
	VGG19	0.891	0.900	0.881	0.890	0.994	0.994	0.994	0.994	0.942 ± 0.035
	NASNetLarge	0.933	0.941	0.924	0.932	0.997	0.997	0.997	0.997	0.955 ± 0.028
	MobileNetV2	0.858	0.735	0.890	0.806	0.989	0.989	0.989	0.989	0.923 ± 0.041
	ResNet50	0.825	0.843	0.805	0.824	0.986	0.986	0.986	0.986	0.905 ± 0.044
ADC-DWI Sequences										
	DenseNet201	0.991	0.992	0.990	0.991	1.000	1.000	1.000	1.000	0.982 ± 0.015
	InceptionV3	0.973	0.975	0.971	0.973	1.000	1.000	1.000	1.000	0.975 ± 0.018
	EfficientNetV2L	0.956	0.959	0.953	0.956	1.000	1.000	1.000	1.000	0.968 ± 0.022
	Xception	0.947	0.950	0.944	0.947	1.000	1.000	1.000	1.000	0.963 ± 0.025
	VGG16	0.930	0.933	0.927	0.930	1.000	1.000	1.000	1.000	0.955 ± 0.029
	NASNetLarge	0.921	0.925	0.917	0.921	0.997	0.997	0.997	0.997	0.949 ± 0.031
	MobileNetV2	0.912	0.916	0.908	0.912	0.994	0.994	0.994	0.994	0.943 ± 0.034
	ResNet50	0.860	0.867	0.853	0.860	0.986	0.986	0.986	0.986	0.918 ± 0.039
	VGG19	0.860	0.867	0.853	0.860	0.986	0.986	0.986	0.986	0.916 ± 0.040

Acc, accuracy; Prec, precision; Rec, recall; F1, F1-score; CV, cross-validation; SD, standard deviation. Cross-validation performed using 5-fold stratified approach.

## 4 Discussion

In this study, the diagnostic performance of Faster R-CNN and nine different deep learning classification models was evaluated using biparametric MR images of 153 patients with histopathologically confirmed PCa or prostatitis. In our study, the Faster R-CNN model achieved 96% accuracy and 98% F1-score for P5 lesions in T2 images, and 90% accuracy and 94% F1-score in ADC-DWI sequences. Among the classification models, InceptionV3, Xception, DenseNet201, and EfficientNetV2L demonstrated excellent performance after data augmentation, achieving 100% values in all metrics. No significant difference was found between the experienced radiologist and the artificial intelligence model in terms of PI-RADS scoring, and both approaches showed high diagnostic success.

### 4.1 Clinical context and diagnostic challenges

Prostate cancer can remain silent for a long time or behave very aggressively. The process of determining tumour behavior stands equally important to the process of making a diagnosis. Cancer seen in the prostate gland can also be very similar to other benign prostate diseases (prostatitis, etc.). For this reason, the diagnosis stage is very critical as it changes the treatment plan. In the PI-RADS v2.1 assessment, a probability estimate on a 5-point scale is made for each lesion in the prostate gland based on the combination of T2A, DWI, and DCE (Dynamic Contrast-Enhanced) MRI findings (25). The diagnostic precision advancement capabilities of artificial intelligence systems represent an essential aspect in this particular scenario.

### 4.2 Technical considerations and imaging protocols

High-resolution T2A imaging serves as a vital sequence for evaluating anatomy and detecting tumors while determining tumor localization and local staging. The diagnostic precision of T2A images stands at a relatively low level on its own. The T2A images present low specificity because hemorrhage alongside chronic prostatitis and scarring and atrophy and radiotherapy and hormone therapy generate hypointense signals that make this method less specific. T2A images require functional sequence integration for better PCa diagnosis because these images exhibit high sensitivity yet low specificity (26). Our research employed a biparametric MR approach because of this. A.E.S Bosaily and colleagues performed an observational study showing that dynamic contrast-enhanced imaging provided no benefit to prostate cancer diagnosis accuracy over T2 and diffusion sequences, so there was no need for prostate MR contrast injection (27).

### 4.3 Comparison with existing literature

Our research findings need to be placed in the context of the wider field of AI applications in medical imaging. Other fields in the literature have utilized Faster R-CNN in numerous studies. The system shows high precision in detecting lung nodules and shows promise for clinical application in diagnosing lung diseases (28). Faster R-CNN reaches 100% accuracy in meningioma tumour classification and 87.5% accuracy in glioma classification while achieving a 94.6% confidence rate in meningioma tumour segmentation compared to MRI (29). The sensitivity of Faster R-CNN reaches 95.64% when detecting lung nodules at a rate of 1.72 false positives per scan (30). Pre-processing of colonoscopy images alongside Faster R-CNN detection enables significant improvement in colon polyp detection rates with an average sensitivity of 91.43% (31).

Specifically in prostate cancer detection, our performance metrics align with or exceed several recent studies. Aldoj et al. (2020) reported that, for the detection of clinically significant prostate cancer defined by biopsy results, their 3D convolutional neural network (CNN) model, trained on multiparametric MRI data from 200 patients, achieved a receiver operating characteristic area under the curve (AUC) ranging from 0.89 (with 88.6% sensitivity and 90.0% specificity) to 0.91 (with 81.2% sensitivity and 90.5% specificity), with an average AUC of 0.897 for the ADC, DWI, and K-trans input combination. Other sequence combinations yielded lower performance. The classification performance was comparable to that of experienced radiologists using PI-RADS, and lesion size or largest diameter did not affect the model’s performance (32). The study by Schelb et al. (2019) showed that a deep learning model (U-Net), trained on T2-weighted and diffusion MRI, had sensitivity and specificity values similar to clinical PI-RADS assessment for the detection of clinically significant prostate cancer. In the external test set, U-Net achieved sensitivities of 96% and 92% at probability thresholds of ≥0.22 and ≥0.33, with corresponding specificities of 31% and 47%, respectively. These results were not statistically different from those obtained by clinical PI-RADS assessment, highlighting that the deep learning approach performed similarly to expert human readers in this context (33).

The DWI and ADC values play essential roles in both lesion characterization and tumour aggressiveness assessment. Prostate cancer replaces tubules by destroying glandular tissue. The ADC values in tumour tissue remain lower than those in the healthy peripheral zones because tumour tissue contains denser cells. The meta-analysis conducted by Lian-Ming Wu and colleagues demonstrated that T2A combined with DWI produced better results than T2WI alone in prostate carcinoma detection with sensitivity at 0.76 and specificity at 0.82 (34). The accuracy range of our study (90-96%) exceeds the reported values in the literature, although direct comparison is limited by differences in patient populations and reference standards.

The research conducted by Mukesh Soni and colleagues demonstrated that SEMRCNN outperformed V-Net and ResNet50-U-Net for prostate cancer detection from MP-MRI with a Dice (similarity) coefficient of 0.654 and a sensitivity of 0.695 (35). The Faster R-CNN model demonstrated superior sensitivity at 0.99 for P5 lesions in ADC-DWI images but this comparison requires careful interpretation because of our limited sample size and different evaluation metrics. Hamide Nematollahi and colleagues conducted a systematic review to determine how different artificial intelligence systems performed in cancer detection tasks. The authors demonstrated that supervised machine learning techniques including deep learning and random forest and logistic regression proved effective for prostate cancer detection through multiparametric MRI (36).

Artificial intelligence models have brought significant progress to prostate cancer detection and segmentation procedures. The non-local Mask R-CNN model achieved 89.5% detection and segmentation accuracy for intraprostatic lesions during histopathological training (37). A. Saha and colleagues demonstrated that a 3D CAD system achieved 83.69% detection sensitivity and AUROC of 0.882 in biparametric MR imaging (38). The AUROC equivalent of approximately 0.94 based on F1-scores indicates competitive performance but we recognize the requirement for direct AUROC calculation in future research. A study conducted in 2024 worked with 400 patients and 11,000 images. The combination of Faster R-CNN and ResNet models produced a modified ResNet which reached 97.4% accuracy in detecting prostate cancer from MR images (39). The accuracy rates between our model (96% for P5 in T2) and their model are similar but their larger dataset provides better evidence for generalizability.

### 4.4 Critical analysis of model performance

Our assessment shows both positive aspects and major weaknesses in our results. Our study results match the outcomes found in previous research conducted in similar contexts. The model shows significant issues in P4 lesion classification because it fails to detect any P4 lesions in T2 sequences and only detects 30% of P4 lesions in ADC-DWI sequences. This represents a major clinical limitation, as P4 lesions carry a 50-70% probability of clinically significant cancer according to PI-RADS v2.1 guidelines (25). The main cause of this outcome stems from both the limited P4 case number (only 2 cases in T2 test set and 10 in ADC-DWI) and the challenging process of measuring these lesions, which requires size-based distinction from P5 lesions. Therefore, it was deemed more appropriate to evaluate P4 and P5 lesions together under the ‘cancer’ label in classification models. When the test results were evaluated, it was observed that the Faster R-CNN model was more effective in analyzing ADC-DWI images and made more predictions based on these images.

### 4.5 Clinical integration considerations

The integration of AI systems into clinical workflows requires careful consideration of multiple factors. Our proposed system would function as a decision support tool rather than a replacement for radiologist interpretation. The workflow would involve: (1) initial AI analysis providing probability scores and anatomical localization, (2) radiologist review with AI assistance highlighting areas of concern, and (3) final interpretation combining AI outputs with clinical context and radiologist expertise. The implementation of this approach faces several challenges because it requires high-quality detailed spatial annotations of training data and methods to handle intra-lesion heterogeneity and weak labeling limitations and sufficient computational resources for full-resolution radiology image analysis (40).

### 4.6 Study limitations

The research contains multiple essential restrictions. The study’s 153 patient sample remains small compared to current multicenter research which reduces the ability to apply our results to other populations. Due to the small number of P4 samples, the model was unable to effectively identify this class, which represents a significant clinical gap as these intermediate-risk lesions require careful evaluation. The model’s performance on an independent dataset remains unverified which creates doubts about its ability to function across different institutions and imaging protocols. The 100% accuracy in some augmented datasets raises concerns about overfitting which needs further investigation because cross-validation results showed 96.8% accuracy. The study did not conduct a prospective clinical trial to evaluate the model’s performance in real-world settings. The study did not evaluate inter-scanner variability because all imaging data was collected from a single 3.0T scanner. The study failed to evaluate how variations in image quality and motion artifacts affect the model’s performance.

### 4.7 Study strengths and contributions

Nevertheless, our study has important strengths. Using the Faster R-CNN architecture, a comprehensive analysis was performed in which zonal anatomical structures were evaluated separately in both DWI-ADC and T2 sequences. The results were directly compared with the evaluations of an expert radiologist with 15 years of experience using nine different deep learning classification models. The sequences that did not need contrast agent achieved high performance levels which provided practical benefits such as shorter scan times and gadolinium risk elimination and cost-effectiveness. Our systematic comparison of multiple architectures provides insights into the relative performance of different deep learning approaches for this specific clinical task.

### 4.8 Future directions

P5 lesions differ from P4 lesions in that they tend to exhibit more locally aggressive behavior. Consequently, the development of models capable of accurately detecting P4 lesions remains a key priority for future research, as such advancements would facilitate the creation of systems that yield more consistent and clinically meaningful predictions. Moreover, the generalizability of current findings requires multi-institutional validation studies to confirm performance across diverse populations, imaging equipment, and imaging protocols.

The clinical utility and impact on patient outcomes of AI-assisted interpretation workflows should be established through prospective clinical trials comparing these workflows with standard diagnostic approaches. Additionally, the clinical value of such systems can be further enhanced by integrating them into electronic health records and clinical decision support platforms. Finally, diagnostic performance may be improved by exploring the synergistic use of radiomic features and deep learning–based methods (41).

## 5 Conclusion

Artificial intelligence models have demonstrated promising diagnostic capabilities in distinguishing prostate cancer from prostatitis using biparametric MR images and may provide valuable support to radiologists as a supplementary tool. The Faster R-CNN model achieved competitive performance compared to similar models in the literature, with the classification models InceptionV3, DenseNet201, and EfficientNetV2L showing excellent performance in our limited dataset. The results show that the experienced radiologist and the artificial intelligence model have similar performance in detecting P5 and prostatitis lesions, which suggests potential clinical utility, but the failure to detect P4 lesions is a critical limitation that must be addressed before clinical deployment.

These findings suggest that artificial intelligence models have potential for integration as clinical decision support systems in prostate cancer and prostatitis diagnosis, but substantial validation work remains necessary. The current evidence supports continued research and development rather than immediate clinical implementation. Future work should focus on addressing the identified limitations, particularly P4 lesion detection and external validation, before these systems can be considered ready for routine clinical use. The path forward requires collaborative efforts between AI researchers, radiologists, and clinicians to ensure that these technologies truly enhance patient care while maintaining diagnostic safety and reliability.

## Representative case examples

Case 1: PI-RADS 5 Lesion (**Figure 3**). The right peripheral zone lesion of the 66-year-old patient received a PI-RADS 5 score from both the radiologist and artificial intelligence system. The T2-weighted images revealed an irregular hypointense lesion with significant DWI b1400 restriction and an ADC map diffusion coefficient of 0.56 × 10^−3^ mm^2^/s. The histopathological examination confirmed prostate adenocarcinoma as the diagnosis (Gleason score 4 + 3 = 7).

Case 2: Prostatitis Lesion (**Figure 4**). The 49-year-old patient received a PI-RADS 3 score from both evaluators for bilateral peripheral zone patchy lesions. The lesions displayed patchy hypointense T2-weighted imaging characteristics and mild DWI restriction and an ADC map diffusion coefficient of 0.96 × 10^−3^ mm^2^/s. The histopathological result was reported as chronic prostatitis.

Case 3: AI Probability Estimation (**Figure 5**). This case demonstrates the AI model’s capability to provide probability estimates for different tissue types. The model correctly identified the cancer with 94% confidence, while also detecting the transitional zone (98%) and peripheral zone (82%) anatomical structures.

## Data Availability

The original contributions presented in the study are included in the article/supplementary material. Further inquiries can be directed to the corresponding authors.
